# Focal bone lesions in hiv-positive patient treated with tenofovir

**DOI:** 10.1186/1471-2334-14-131

**Published:** 2014-03-06

**Authors:** Davide Mangioni, Alessandra Bandera, Antonio Muscatello, Nicola Squillace, Cinzia Crivellaro, Luca Guerra, Cristina Messa, Andrea Gori

**Affiliations:** 1 Division of Infectious Diseases, Department of Internal Medicine, San Gerardo Hospital, University of Milano-Bicocca, Monza, Italy; 2 Department of Nuclear Medicine, San Gerardo Hospital, University of Milano-Bicocca, Monza, Italy

**Keywords:** HIV, Tenofovir, Bone lesions

## Abstract

**Background:**

Tenofovir is a widely used antiviral drug for the treatment of HIV and HBV infection. Although its side effects on renal function and bone metabolism are well known, there are no reports on focal bone lesions caused by this drug. Our case suggests this new, unusual but important scenario.

**Case presentation:**

We report on a 46-year-old HIV-positive man treated with an antiretroviral regimen containing tenofovir who suddenly developed localized inflammatory bone lesions. The examinations performed ruled out all the disorders commonly associated with this clinical pattern, and the patient’s conditions improved only after the suspension of tenofovir.

**Conclusions:**

The case study suggests a rare but severe adverse event, which should be taken into account when physicians treat HIV-positive patients with focal inflammatory bone lesions

## Background

Tenofovir (TDF) is an antiviral drug widely used as first-line therapy in HIV infection and prophylaxis as well as in HBV infection [1]. It is frequently chosen for its efficacy and its ease of use in the once-daily single-tablet regimens [1,2]. However, it has been clearly associated with renal toxicity [3] and with a decrease in Bone Mineral Density (BMD) [4]. Metabolic bone diseases are often an issue in patients with HIV infection: the prevalence of osteoporosis-associated fractures has been found to be 60% higher in HIV-infected patients as compared with HIV-uninfected persons, with a 6.4 fold increased odds of osteopenia and 3.7-fold increased odds of osteoporosis [5]. Important pathogenic roles have been identified in the virus itself, in the immune activation triggered by HIV [6], in individual risk factors (such as sex, age, low BMI, smoking, alcohol abuse, HCV coinfection) and in drug toxicities, particularly from TDF [7]. Nevertheless, there is no report in the literature about focal bone damages in HIV-infected patients due to this drug.

## Case presentation

We report on a 46-year-old homosexual man who has been HIV seropositive since 1994, with a history of multiple undiagnosed episodes of osteo-articular pain with no elective localizations and always resolving after the assumption of non-steroidal anti-inflammatory drugs. The patient's HAART regimen was tenofovir-entricitabine and lopinavir/ritonavir since 2008. He was also treated with fenofibrate and amisulpride at low dosage (50 mg 1 tablet QD). His viral load was undetectable since 2003, and the CD4+ T cell count 466 cells/μL in the last blood test before the time of this report. In April 2011 the patient performed lumbar spine and femoral neck DEXA (Dual-Energy X-ray Absorptiometry) for osteopenia screening. Lumbar spine DEXA showed: BMD = 1.043 g/cm^2^, T-score = -0.4, Z-score = -0.2; in the femoral neck DEXA: BMD = 0.868 g/cm^2^, T-score = -0.5, Z-score = 0.2. All of these values were within their normal limits.

**Figure 1:**
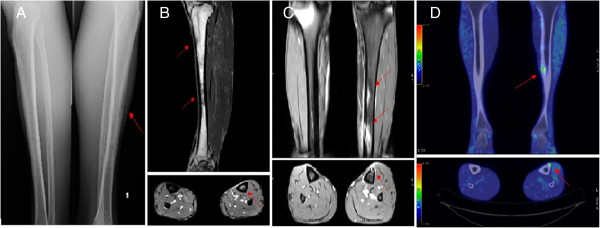
**Morphological and functional imaging of the patient's bone lesions. A**: Anterior-posterior x-ray image: focal bone loss of uncertain significance localized in the proximal and middle diaphysis of the left tibia. **B**: Sagittal and axial NMR images: focal alterations of the left tibia, both in the medullar and in the cortical portion, probably related to an inflammatory process. **C**: Coronal and axial NMR images: contrast-enhanced cortico-subcortical abnormalities in the middle-distal diaphysis of the left tibia. **D**: PET/CT fused images (coronal and axial): increased uptake of FDG in the diaphyseal cortex of the left tibia associated with structural alterations probably connected to inflammatory results.

Two months later, the patient came to our Clinic after 4 weeks of remittent fever with peaks of 38°C and signs of inflammation and progressive joint pain in the left lower limb. He was hospitalized and we carried out first level examinations: lower limbs x-ray and doppler ultrasound ruled out bone fractures and deep vein thrombosis. Multiple blood cultures resulted negative, while the markers of inflammation were elevated (ESR: 68 mm/h, normal range: 0-15; CRP: 9.24 mg/dL, normal range < 0.50). Interestingly, the x-ray images showed a focal bone loss localized in the left tibia [Figure 1 Panel A]. Subsequently, the following laboratory test were performed: autoimmunity screening panel (Anti-Nuclear, Anti-Ro/SS-A, Anti-La/SS-B, Anti-smooth muscle, Anti-mitochondrial, Anti-liver kidney, and Anti-neutrophil cytoplasmic antibodies), serologies for HCV, Rickettsiae, Coxiella bruneti, Brucella, Cryptococcal antigen, and Quantiferon TB-Gold. All the blood tests resulted negative. Notably, most of the patient's values of renal and tubular function (creatinine, urine inorganic phosphate levels, urine calcium: urine creatinine ratio) were within their normal ranges. So were most of the markers of bone metabolism (see Table 1); only the levels of 25-OH vitamin D3 were below the physiological limits (9.4 ng/mL, normal values > 30 ng/mL).

**Table 1:** Principal markers of bone metabolism at the time of hospitalization and at the 4-month follow-up

	**Normal range**	**June 2011**	**October 2011**
Total calcium, plasma (mg/dL)	8.5 – 10.3	9.1	9.4
Inorganic phosphate, plasma (mg/dL)	2.0 – 5.0	2.9	2.2
Bone alkaline phosphatase, serum (mcg/L)	6 – 30	19.2	11.2
Parathormone (PTH), plasma (pg/mL)	15.0 – 65.0	57.4	62.2
Osteocalcin, serum (ng/mL)	14 – 42	21	13
Beta-cross laps, plasma (pg/mL)	< 584	336	256
25-OH Vitamin D3, serum (ng/mL)	> 30	9.4	24.9

We then planned a medication wash-out, together with more detailed investigations: I) a lower left limb nuclear magnetic resonance (NMR) without contrast enhancement demonstrated focal alterations of the tibial diaphysis, likely linked to an inflammatory process [Figure 1 Panel B]; II) a contrast enhancement NMR of the lower extremities revealed multiple millimetric contrast-enhanced cortico-subcortical abnormalities in the left tibia diaphysis [Figure 1 Panel C]; III) a positron emission tomography - computed tomography (PET-CT) with [18 F] FDG, performed on June 2011, confirmed the presence of multiple areas of increased uptake in the diaphyseal cortex of the left tibia, that were not interpreted as neoplastic lesions but rather as inflammatory results. [Figure 1 Panel D]; IV) multiple bone biopsies (which showed focal necrosis and remodeled lamellar bone tissue with interlamellar spaces occupied by fibrosis and round-oval elements not otherwise specified) were examined by two different and independent pathologists, definitely excluding a malignant disease.

The patient's clinical and biochemical conditions gradually improved, so we decided to reintroduce a new antiretroviral therapy with lamivudine-abacavir and lopinavir/ritonavir, opting for a TDF-free regimen. The patient underwent a biochemical follow-up 4 months after the hospitalization (Table 1), but refused further imaging evaluations we proposed. He eventually recovered a good viro-immunological profile and did not complain about any osteo-articular pain thereafter.

## Conclusions

The effect of tenofovir on the bone metabolism, especially when used in combination with protease inhibitors, is well demonstrated [8]. This iatrogenic impairment occurs on a system already damaged by the persistent immune activation due to HIV and by associated risk factors [4-7]. The most frequent consequence is osteoporosis. In our case, however, despite the evident condition of hypovitaminosis D, the normality of all the other laboratory tests (especially PTH and beta-cross laps, important markers of bone remodeling) and the DEXA examination, make the diagnosis of osteoporosis at least unlikely. So were the hypotheses of secondary hyperparathyroidism (given the values of the markers of bone metabolism, Table 1) and of a tubular kidney disease, as we could gather from the results of renal and tubular function markers. In addition, the morphological and functional imaging and the clinical presentation clearly suggest localized inflammatory lesions.

The patient's positive response to the medication wash-out and the absence of a disease relapse after the reintroduction of HAART without tenofovir led us to assume a central role of this drug in the onset of the bone lesions. Our hypothesis is supported by the strong clinical evidence on the effects of tenofovir on the bone metabolism, and by the pathophysiology mechanisms of TDF (which directly interacts with the osteoblast activity) [1,4,5,9]. Yet the reason why the patient, with a stable viro-immunological situation, showed this clinical picture remains unclear. It can be thought that it happened when the "osteoimmunological balance” in the immuno-skeletal interface [6] had been altered.

We consider, therefore, our report noteworthy: this possible side effect of tenofovir, although unusual, should be taken into account in the differential diagnosis of an HIV-positive patient with focal bone lesions.

## Consent

Written informed consent was obtained from the patient for publication of this Case report and any accompanying images. A copy of the written consent is available for review by the Editor of this journal.

## Abbreviations

BMD: Bone mineral density; DEXA: Dual-energy x-ray absorptiometry; CRP: C-reactive protein; ESR: Erythrocyte sedimentation rate; HAART: Highly active antiretroviral therapy; HBV: Hepatitis B Virus; HCV: Hepatitis C Virus; HIV: Human Immunodeficiency Virus; FDG: Fluorodeoxyglucose; NMR: Nuclear magnetic resonance; PET-CT: Positron emission tomography - computed tomography; PTH: Parathormone; QD: Quaque die; TDF: Tenofovir.

## Competing interests

The authors declare that they have no competing interest.

## Authors’ contributions

All authors contributed equally to this work.

## Pre-publication history

The pre-publication history for this paper can be accessed here:

http://www.biomedcentral.com/1471-2334/14/131/prepub
